# Attention and working memory: two basic mechanisms for constructing temporal experiences

**DOI:** 10.3389/fpsyg.2014.00880

**Published:** 2014-08-14

**Authors:** Giorgio Marchetti

**Affiliations:** Mind, Consciousness, and Language Research NetMilan, Italy

**Keywords:** mental time travel, language, thought, attention, working memory, duration, past, future

## Abstract

Various kinds of observations show that the ability of human beings to both consciously relive past events – episodic memory – and conceive future events, entails an active *process of construction*. This construction process also underpins many other important aspects of conscious human life, such as perceptions, language, and conscious thinking. This article provides an explanation of what makes the constructive process possible and how it works. The process mainly relies on attentional activity, which has a discrete and periodic nature, and working memory, which allows for the combination of discrete attentional operations. An explanation is also provided of how past and future events are constructed.

## INTRODUCTION

Tulving (1972) noted that one of the most fascinating achievements of the human mind is its ability to mentally travel through time. We can relive experiences by thinking back to situations that occurred in the past; likewise, we can mentally project ourselves into the anticipated future through our imagination, daydreams and fantasies.

Time travel is just one of the main *forms* that consciousness can assume. As phenomenologists have shown (Gallagher and Zahavi, 2008), there are various modes of givenness of objects and events, that is, ways in which objects and events appear to us. It is possible for one and the same object or event to appear in a variety of other different ways: from this or that perspective, linguistically represented, dreamt, imagined, perceived, wished for, or feared.

Each of these forms of consciousness possesses its own phenomenal quality: perceiving an object feels different from imagining the same object, which in turn feels different from remembering it. Despite the phenomenal differences, it can be shown that most of these various forms of consciousness depend on *constructive* processes based on some common mechanisms. This is suggested for example by the observation that they can be “added” to every conscious content, in the sense that the same conscious content can be experienced in one or the other form of consciousness by means of just mentally processing it through one or the other form of consciousness (for example, we can experience the same event as occurring either in the past or future by means of just mentally adding to it the temporal dimension of past or future, respectively, Nyberg et al., 2010).

In this article I will provide psychological and neurophysiological data to substantiate the idea that some of the most important forms of consciousness – episodic memory, episodic future thought, perception, language, and conscious thinking – are based on constructive processes based on two common mechanisms: attention and working memory. The sections *Time travel* and *Perception, language and conscious thinking* will provide evidence supporting the general idea that these forms of consciousness are the result of an active process of construction performed by the subject. In the section *The underlying mechanisms of constructive processes: attention and working memory* I will try to show that the constructive processes underpinning the various forms of consciousness, rely on two common and fundamental mechanisms: attention and working memory. Finally, the section *Variously using attention and working memory yields various construction processes* will show how constructive processes can yield various forms of consciousness in general, and specifically how the conscious experience of temporality is constructed.

## TIME TRAVEL

### EPISODIC MEMORY

Episodic memory – the ability to consciously relive personal past events (Tulving, 1985) – is an active process of construction, rather than a faithful re-enactment of the past (Rosenfield, 1988; Suddendorf et al., 2009). Various kinds of observations show this.

As Schacter (1999) has shown, memory generally suffers from different types of “sins,” which can be classified into three main categories. The first category involves forgetting: memory of facts and events typically becomes less accessible over time, even when we deliberately search our memory in an attempt to recall a specific fact or event; memory of facts and events is affected by attention: when insufficient attention is paid to a stimulus at the time of encoding or retrieval, forgetting is likely to occur, as the phenomenon of change blindness shows (Simons and Levin, 1997); even when a fact or event has been deeply encoded, and has not been lost over time, it may occasionally be temporarily inaccessible (one of the most common and frequent instances is the tip-of-the-tongue state). The second category involves distortion or inaccuracy: even if people may remember correctly a fact from a past experience, they can misattribute it to a wrong source; suggestions or misleading questions that are made when one is attempting to recall an experience, may alter the recollection of the original event; memories can be biased and distorted by expectations, beliefs, current knowledge, etc. The third category refers to pathological remembrances: facts and events, such as traumatic events, that we cannot forget even if we wish we could. While, on the one hand, these “sins” demonstrate the occurrence of memory distortions, on the other hand, they clearly support the idea that memory is not so much a literal replay of the past as a complex process of assembling and integrating several bits of information.

Moreover, as Tulving (1972) already noted, different memory systems differ in their susceptibility to transform and lose stored information, which further complicates the picture drawn by Schachter. For example, forgetting appears to be more readily produced in episodic memory than in semantic memory. Since information in the former is always temporally dated, and can only be retrieved if the retrieval cue accurately specifies its temporal date, interference with temporal coding may render access to it difficult or impossible. On the contrary, information in semantic memory, being usually encoded as part of a rich multidimensional structure of concepts and their relations, is better protected by such embeddedness from interference by other inputs.

The phenomenon of déjà-vu, in which we have the experience of reliving a past event in the absence of an actual memory, shows that the sense of “pastness” of the event is not inherent in the memory itself and that it depends on a process of active construction.

Conversely, merely using a memory representation of a prior event is not sufficient to ensure the subjective experience of remembering it: as Suddendorf et al. (2009) notice, semantic memory allows us to know where and when we were born but this does not suffice to consciously relive that moment.

### EPISODIC FUTURE THOUGHT

Episodic future thought is the ability to simulate specific personal episodes that may potentially occur in the future (Szpunar, 2010). In many cases, it is patent that episodic future thought involves a process of active construction of events that have not yet occurred, such as when future thought depends on a (novel) recombination of episodic details (whether of perceptual or imaginal source) into a hypothetical event. This is what experiments performed using the word-cueing paradigm (Galton, 1880; Crovitz and Schiffman, 1974) generally reveal: when participants are given a word cue and are asked to use it to mentally generate personal future details, they tend to imagine themselves in the context of familiar settings and people (D’Argembeau and Van der Linden, 2004).

It should be noted, however, that not always does future thought depend on a recombination of episodic details. Sometimes, future thoughts and episodes may be constructed without the need to necessarily rely on the contents of specific episodic memory *per se*: a general, abstract knowledge of the context, environment or situation is sufficient to imagine how things can evolve in future (Szpunar, 2010).

The constructive process involved in episodic future thought is highlighted by experiments such as Nyberg et al.’s (2010). Nyberg et al. (2010) scanned well-trained subjects using fMRI during experimental tasks that require the capacity to be aware of subjective time (chronesthesia), such as remembering a recent short walk along a familiar route or imagining a future short walk along the same route. Brain activity during these tasks was compared with activity during a matched task that does not require chronesthesia: the subjects were instructed to take a mental walk through the same route in the present moment, without any thoughts about specific personal past or future happenings. By contrasting the remembering and imagining tasks with the mental walk task, Nyberg et al. (2010) could measure brain activity correlated with “pure” conscious states of different moments of subjective time. They reported that the left lateral parietal cortex, as well as the left frontal cortex, cerebellum, and thalamus were preferentially engaged as the subjects thought about taking walks in the past or future as compared to taking the same walk in the present moment.

This study, while revealing which brain regions are specifically related to the capacity to be aware of subjective time, also clearly shows that an event (such as taking a short walk from a certain point to another) can alternatively be experienced as occurring in the past or future by means of just mentally adding to it the temporal dimension of past or future, respectively. That is, an event that is otherwise lived as present may become a past or future event if we actively construct it as such, by mentally adding to it a specific past or future temporal quality. (Incidentally, it should be noted that I use the term “construction” here in a wider sense than; Nyberg et al., 2010. While they use it to refer to the sole process of constructing the scene of walking, my usage also includes the process of adding a temporal dimension, and hence of constructing the subjective experience of time).

That subjective time requires to be mentally constructed in order to be consciously experienced, is further evidenced by all those processes that, despite dealing with future events, can take place independently from being experienced as occurring in time. For example, Kwan et al.’s (2012) study shows a clear dissociation between imagining and knowing about the future. K. C., a person with episodic amnesia and unable to imagine future experiences, can still value future reward and make a decision about the future in a way comparable to healthy subjects, despite being unable to construct the details of either past or future events. Therefore, the ability to make decisions about the future is dissociable from the ability to imagine one’s possible future. It should be noted that the decision-making strategy of healthy individuals – like those of the controls in Kwan et al.’s (2012) study – usually involves future-oriented imagery (see also Peters and Büchel, 2010, who show how the ability to imagine one’s possible future modulates future decision-making). This is additional evidence that humans can add a temporal dimension to a process that otherwise occurs outside of any temporal experience.

Another case in which the capacity to deal with future events does not necessarily depend on the capacity to simulate or imagine future events is anticipatory behavior. As observed by Suddendorf and Corballis (1997), instincts, such as hibernation, provide a mechanism for dealing with environmental changes without the need for the organism to actually imagine the future (hibernators prepare for winter even if they have not experienced that season before). This parallels the distinction between episodic and other memory systems, which explains how the past can influence behavior without necessarily involving mental time travel into the past.

To summarize, both episodic memory and episodic future thought are best conceived as constructive processes, rather than literal records of the past or a projection in the future of carefully represented episodic memories, respectively. This common characteristic is further highlighted by research findings from neuroimaging, neuropsychology, and clinical psychology that have shown a close relationship between episodic memory and episodic future thought: (a) neural regions believed to underlie the retrieval of personal memories are similarly engaged by episodic future thoughts; (b) damage to these regions is associated with impairments of both remembering and episodic future thought; (c) patients characterized by poor episodic memory exhibit a concurrent inability to imagine their future in a vivid way (for a review, see Szpunar, 2010; however, it should be noted that differences between the two processes exist as well: see Schacter et al., 2012).

## PERCEPTION, LANGUAGE, AND CONSCIOUS THINKING

Episodic memory and episodic future thought are not the only forms of human consciousness based on an active construction process. Construction processes also underlie many other important forms of human conscious life, such as perceptions, language, and conscious thinking.

### PERCEPTION

Just as memories are not a faithful re-enactment of the past, so too perceptions are not a faithful representation of a world specified prior to, and independently of, the activity of the subject. Daily experiences and perceptual illusions show how situational and contextual factors, as well as expectations, education and emotions, shape our perceptions. If we consider the perception of space alone, we see that different sense-organs generate different spatial perceptions of the same object: a piece of rosemary stuck in a tooth feels enormous until it is felt with your fingers. Likewise, emotions change our perception of space: in despair, for example, there is nowhere to go, the sky closes in, actions seem pointless, and the ordinary depths of the world are transformed (Morris, 2004). The perception of the dimension of the same object changes in relation to the different context in which it is placed: a piece of furniture may seem smaller when seen in a shop than when seen inside our house. Finally, the perception of space also changes with age: what seemed to be big, large and high in size when we were children (for example, the house in which we were born) may appear to be small, narrow and low as adults.

These examples clearly show that perceptions are not a passive duplication of a ready-made world, but the result of an active process of construction performed by the subject. Evidence of this process of construction is provided by various kinds of findings. Neural data show that the complex representations generated by the visual system are built out of distinct streams of processing: an occipitotemporal ventral stream in which cells are sensitive to information pertaining to the identity of objects and an occipitoparietal dorsal stream in which cells are sensitive to spatial information (Ungerleider and Mishkin, 1982). Within each of these streams, information diverges further: there are distinct streams for processing color, shape and motion, and multiple representations of space within the posterior parietal cortex. Moreover, activity in the visual system can be modulated by attention (Treue, 2001; Boynton, 2005; Carrasco, 2011), which allows the organism to adequately cope with contextually and behaviorally relevant information.

Psychological phenomena such as the continuous wagon-wheel illusion (VanRullen and Koch, 2003; Simpson et al., 2005; VanRullen et al., 2005, 2006), perceived causality (Shallice, 1964) and apparent simultaneity (Hirsh and Sherrick, 1961) and neurophysiological observations (Fingelkurts et al., 2010) reveal that our experience of the surrounding world as a continuous, seamless flow of information, is actually the result of the combination and assembly of distinct processing epochs. Studies on the phenomenology of temporal perception of events (Pöppel, 1997, 2004; Wittmann, 2011) show that one can identify at least three different levels at which successive events are fused to form distinct subjective experiences, each possessing its own specific qualitative characteristics. According to Wittmann (2011), on a first, basic level there is the “functional moment,” an elementary temporal building block of perception in the range of milliseconds, which has no perceivable duration because individual events are processed as co-temporal and the temporal order of events is not detected. On a second level, successive functional moments are grouped on a time scale of up to around 3 s, yielding the “experienced moment,” where events are perceived as occurring in an extended now. Within the experienced moment, successive events are strongly and orderly bound together: when listening to a metronome at moderate speed, we do not hear so much a train of individual beats, as perceptual gestalts having an accent on every *n*th beat, such as “1–2, 1–2” or “1–2–3, 1–2–3.” If, on the contrary, the metronome is too fast, we experience a fast train of beats that does not contain any temporally ordered structure of distinct events. Likewise, if the metronome is too slow, we perceive only individual beats which are not related to each other. A third level of integration exceeding about 3 s leads to “mental presence,” a sequence of experienced moments enclosed within the temporal window of a unified experience of presence, which enables the continuous awareness of oneself as presently perceiving and acting within an environment (Fingelkurts and Fingelkurts, 2014). In sum, whereas the duration of the functional moment is not perceived at all, an experienced moment is perceived as happening now, for a short but extended moment. On the contrary, mental presence involves the experience of a perceiving and feeling agent within a window of extended present.

Despite the fact that we experience the world surrounding us as a continuous, seamless flow of information, as we do when watching a movie, we actually extract and process information in distinct moments, similar to the snapshots of a camera. This observation is supported by empirical findings. For example, Latour (1967) found that the visual threshold for detecting a flash of light varied periodically in the few milliseconds preceding the onset of an eye saccade, and that the visual threshold for detecting two flashes displayed successively varied periodically as a function of the time interval between them. This data can be interpreted as evidence of periodical processing on the grounds that the probability of detecting a brief stimulus varies as a function of the state of the process: during a “no-processing” state, the detection rate of the stimulus decreases, whereas it increases during the “processing” state. Periodicities were also observed in reaction time distributions (Venables, 1960; Dehaene, 1993), which can be explained in the following way: a stimulus with an onset occurring during a “no processing” state must wait until the next processing state in order to be processed, which leads to a longer reaction time.

In a face identification task, Blais et al. (2013) modulated the signal-to-noise ratio of faces through time, so that at some moments visual information was available, whereas at other moments no information was available, and analyzed how different temporal profiles of signal-to-noise ratio impacted face identification performance. The aim was to test the hypothesis that visual information would be sampled periodically. The underlying assumption was that if the information useful for the task is available at the right moment, the participant is more likely to respond correctly. In contrast, if the information useful for the task is not available at the right moment during the processing, then the participant is less likely to respond correctly. The results of Blais et al.’s (2013) experiments show the existence of a discrete sampling of visual processing, operating at a rate of about 10–15 Hz. Moreover, their findings support the view that this periodical mechanism synchronizes with the visual stimulation.

Data from electrophysiological recordings show that electrical neural oscillations could provide the physiological basis of periodic perceptual phenomena. Varela et al. (1981) found a close correlation between the perception of apparent simultaneity and the alpha phase at which stimuli are presented: two flashes of light that always have the same stimulus onset asynchrony are judged to be simultaneous when presented at one particular phase, but sequential when presented at the opposite phase. Busch et al. (2009) and Mathewson et al. (2009) show that the phase of ongoing oscillations influence whether a stimulus is perceived at all, which indicates that the visual detection threshold is not constant over time but fluctuates along with the phase of spontaneous electroencephalogram (EEG) oscillations. Drewes and VanRullen (2011) show that the prestimulus oscillatory phase modulates human saccadic reaction time. Investigating the role of prestimulus phase coupling on visual perception in an attentional blink paradigm, Kranczioch et al. (2007) found that low levels of prestimulus alpha phase coupling predict correct perception of the second target stimulus, whereas high levels of prestimulus alpha phase coupling predict a miss of the second target stimulus. Doesburg et al. (2009) showed that perceptual switching during binocular rivalry is time-locked to gamma-band synchronizations which recur at a theta rate, indicating that the onset of new conscious percepts coincides with the emergence of a new gamma-synchronous assembly that is locked to an ongoing theta rhythm. Doesburg et al. (2009, ibid., p. 2) infer that “only one truly discrete perceptual experience may exist within a single theta cycle, and that the emergence of new perceptual experiences may be time locked to a particular phase of ongoing cortical theta rhythms.”

There is evidence that the amplitude of prestimulus oscillations in the alpha range also significantly affects the perceptual outcome. Van Dijk et al. (2008) found that contrast-discrimination ability is modulated by prestimulus alpha power: an increase in posterior alpha power correlates with a decrease in discrimination ability. By directly stimulating visual area via short transcranial magnetic stimulation (TMS), Romei et al. (2010) tested whether oscillation in the alpha band causally shapes perception, relative to control stimulations in the theta and beta bands: they found that occipital and parietal TMS at alpha frequency impairs target visibility in the visual field contralateral to the stimulated hemisphere and enhanced it ipsilaterally.

It still remains to be defined which parameter of neural oscillations – amplitude, phase consistency, or phase coupling – predicts periodicity in perception better than others (Hanslmayr et al., 2011). Likewise, it still remains unclear why different frequencies correlate with different periodic perceptual phenomena. Various hypotheses could be supported. For example, one can think that the sampling frequency could vary as a function of the kind of stimulation, synchronizing with stimulation, or that it could evolve without synchronizing with the external world, as a passive ongoing, random oscillation (Blais et al., 2013). Despite all these open questions, however, the bulk of current studies clearly points to the idea of a discrete sampling of perceptual processing.

### LANGUAGE AND CONSCIOUS THINKING

There is no doubt that the human capacities that most apparently involve an active construction process are language and conscious thinking. Language allows us to combine the single words and thus convey vastly, literally infinite new meanings and conscious experiences. The power of language as a unique and specialized tool in connecting objects and events is exemplified by the strong connection between language and the ability to encode and represent the order of discrete elements occurring in a sequence (sequential learning; Conway and Christiansen, 2001). Likewise, conscious thinking develops and evolves thanks to the combination of ideas, concepts, images, memories etc. As Baumeister and Masicampo (2010, p. 956) observe: “conscious thinking and speech involve a process of actively combining concepts to make something that may have additional, unforeseen, newly emergent properties.”

As extensively showed by the works of linguists, logicians, and philosophers, the combinatorial property of language and conscious thinking relies on some specific relational units, whose function is to tie together two or more semantic elements, be they simple words, other relational units, complex thoughts, or else. Scholars have variously identified and termed these relational units. Sapir (1921) named them “relational concepts” and classified them as concrete and pure. Ceccato (1972; see also Ceccato and Zonta, 1980) termed them “correlators.” Linguistically, correlators are designated by prepositions, conjunctions, cases (genitive, dative, etc.) and the implicit correlator (Benedetti, 2011). Other examples of correlators are the mathematical and logical operators. As Benedetti’s (2009, 2011) in-depth analysis shows, correlators are themselves constituted by sequences of elemental mental operations (among which those of attention play a key role) that are assembled and chunked together by means of working memory and procedural memory. The assembling and chunking processes are supposed to be supported by analogous processes at the neurophysiological level within the nested hierarchy of brain operational architecture (Benedetti et al., 2010; Fingelkurts et al., 2010, 2013).

The constructive character of language is further evidenced by its intrinsic periodicities. Speech is not produced in a continuous, uninterrupted flow but in spurts. Spurts reflect not only a biological necessity (that is, the speaker’s need to replace the air that he expels from his lungs when he produces speech sounds) but also the basic functional segmentations of discourse. Chafe (1994) refers to these segments of language as “intonation units.” Intonation units, whose size averages one to four words, can be identified on the basis of a variety of criteria, among which are pauses or breaks in timing, acceleration and deceleration, changes in overall pitch level, etc. Each intonation unit “verbalizes a small amount of information which, it is plausible to suppose, is part of the speaker’s model of reality on which his or her consciousness is focused at that moment. In a socially interactive situation it is the portion on which the speaker intends that the listener’s consciousness be focused as a result of hearing the intonation unit. This limited activation allows a person to interact with the surrounding world in a maximally productive way, for it would hardly be useful to activate everything a person knew at once” (Chafe, 1994, ibid., p. 29).

Similarly, Duncan (2013) explains the combinatorial nature of human thought and, more in general, of cognition as the most appropriate and adaptive answer to the complex problems posed by the environment. It allows us to flexibly address very complex problems through the solution of simpler sub-problems. As made clear by Artificial Intelligence studies, the most effective way of solving complex problems is by decomposing them into simpler components (see also Fingelkurts et al., 2012). If all the aspects of a problem were considered at once, the search space of possible alternative solutions would simply be too large and unconstrained, yielding too many concurrent, suboptimal choices. “Effective cognition requires a series of selections from this space, each defining a subproblem of relevant inputs, actions, and potential achievements. Often these will be organized hierarchically, so that each subgoal or task is divided further into subgoals of its own” (Duncan, 2013, ibid., p. 36).

## THE UNDERLYING MECHANISMS OF CONSTRUCTIVE PROCESSES: ATTENTION AND WORKING MEMORY

As we have seen, disparate forms of consciousness such as time travel, perception, language, and conscious thinking are all based on an active construction process. As revealed by research reviewed in the previous sections, this construction process is implemented at various levels and underpinned by various mechanisms. Given the commonality and close relations between these forms of consciousness (language influences perception and *vice versa*, conscious thinking and language show extensive commonalities, mental time travel is heavily based on perceived objects and events), it is legitimate to ask whether the various forms of consciousness are underpinned by a common construction process or, at least, whether the constructive processes underpinning them are based on some common mechanisms.

My analysis (Marchetti, 2010) reveals that (1) different forms of consciousness are produced by different construction processes, and (2) two mechanisms implement the different construction processes that underpin most of the different forms of consciousness: these two mechanisms are attention and working memory. Attention ensures the selection of basic elements (or pieces of information); working memory ensures that the selected elements are maintained active during processing and assembled. Various forms of consciousness result from the different ways that attention and working memory operate, that is, from the different ways of selecting, maintaining and assembling basic elements.

Some form of attention is always necessary to produce consciousness (Bor and Seth, 2012; Marchetti, 2012), even though the former does not always produce conscious outcomes. Working memory, while being necessary for most of our conscious experiences, does not seem to be always necessary: very simple and basic perceptions, but also more complex forms of perception involving top-down attention control, do not require working memory. Kane et al. (2006) showed that even in contexts (such as command search task) in which subjects have to endogenously control visual attention by moving it strategically though search arrays, the high- and low- working memory subjects performed equivalently: that is, even visual search tasks that present minimal demands to actively maintain or update goal-relevant information, but which are still difficult and involve top-down attention control, are independent of working memory.

The combined working of attention and working memory is necessary to produce most of our conscious experiences. However, attention and working memory are not always sufficient: most forms of consciousness also need other components, such as long term memory, semantic memory, sense-organs, and somatosensory organs, and what I call the schema of self (Marchetti, 2010).

Before examining how attention and working memory implement construction processes, I will firstly present evidence that attention operates in a periodic, pulse-like manner, thus providing a plausible explanation for the periodicities observed in perceptions and language, as well as for the selections performed in conscious thinking and time travel. Secondly, I will briefly consider the neurophysiological bases of working memory, and exemplify the role it plays in a form of consciousness, episodic future thought.

### ATTENTION

Psychologists have long studied attention as a mechanism capable of coping with the limits of our sensory, perceptual and memory systems in managing the flow of information with which we are constantly confronted. By allowing for the selection of the information, attention reduces the input to a manageable amount: it isolates and amplifies pieces of information, which can be variously combined to yield theoretically infinite chains of constructs. As observed by VanRullen et al. (2007), overt periodic sampling of the environment is a ubiquitous property of sensory systems (saccades in vision, sniffs in olfaction, whisker movements in rat somatosensation, and even electrolocation in the electric fish) and attention might have evolved from these periodic processes as a more economical means of covertly sampling endogenous representations.

Moreover, as argued by Duncan (2013, p. 36), attention proves to be an effective tool in dealing with the complex problems posed by the environment: in fact, it allows for the segmentation of the flow of information into “attentional episodes,” each episode admitting into consideration only the contents of momentary, focused subproblems. More specifically, attentional discrete processing has various advantages from a purely computational point of view. According to Buschman and Miller (2010), restricting computations to discrete windows of time would: (a) ensure that informative spikes occur with the temporal precision that is both necessary for integration by downstream neurons and for spike-timing dependent plasticity; (b) act to stabilize and organize the neural network and its computations: periods of inhibition may act to “reset” the network to a base state, effectively limiting the number of states that neurons could obtain; (c) allow for easier coordination of processing within and between brain regions, by providing a specific moment at which information must be available for computation in a specific region, and at which the outcome of the computation is available.

Finally, the attentional selection process has the side effect of creating new experiential dimensions on top of the ones from which they originate. By selecting and combining otherwise unrelated elements, we can imagine and simulate new events, scenarios and conditions that we would have never consciously experienced if we had not been endowed with selective and constructive capacities. As observed by Baumeister and Masicampo (2010, p. 958), the full power of human consciousness consists in using the mental capacity for constructing sequential thoughts to conduct simulations during wakefulness, without relying on sensory input.

Although the working of attention can be theoretically conceived as an uninterrupted, continuous process, which rapidly switches between different targets, evidence seems to favor the hypothesis that attention operates in a periodic, pulse-like manner. VanRullen et al. (2007) found that attention, even when focused on a single target location, samples information periodically like a blinking spotlight. Moreover, by analyzing the correlation between detection performance for attended and unattended stimuli and the phase of ongoing EEG oscillations, Busch and VanRullen (2010) showed that detection performance for attended stimuli actually fluctuated over time along with the phase of spontaneous oscillations in the θ (≈7 Hz) frequency band just before stimulus onset. This fluctuation was absent for unattended stimuli. This pattern of results suggests that attention in fact exerts its facilitative effect on perception in a periodic fashion. The alpha phase plays a crucial role in the attentional blink phenomenon (Hanslmayr et al., 2011). Doesburg et al.’s (2008) findings support the view that gamma-band synchronization is the mechanism that implements the selective properties of attention, its integrative properties, and the special relationship between attention and consciousness. Landau and Fries’ (2012) study shows that selective attention samples stimuli in a rhythmic way. Further evidence of the link between attention and periodic brain processes is provided by studies in which oscillatory brain responses are entrained by periodic stimulation (Jones et al., 2002).

An interesting theoretical framework has been proposed by Fingelkurts and Fingelkurts (in press) as to the neural mechanisms of attention. Fingelkurts et al. (2009, 2010, 2013) conceptualize attention within their theory of the operational architectonics (OA) of brain and mind functioning. According to this theory, involuntary (bottom-up) attention arises as a result of self-organized formation of neuronal assemblies whose operations are divided by rapid transients (so called RTPs in the brain oscillations) that signify the breakpoints of attention leading to an attentional disengagement, shift, and/or allocation to a new operation. Fingelkurts and Fingelkurts (in press) further suggest that the duration of these operations is determined by external stimuli and modulated by arousal as well as affective reinforcement. Voluntary (top-down) attention emerges as a result of binding of multiple operations responsible for sensory percepts or motor programs in a context-dependent way as a function of a saliency, prior knowledge and expectancies. During this process, the ever-changing and multiform stream of cognition and conscious experiences is somehow “frozen” and “classified,” thus leading to the phenomenological experience of acts or moments of focused attention in which our consciousness is kept focused as a mental magnifying lens at the attended object or scene. According to Fingelkurts and Fingelkurts (in press), the skill to voluntarily focus attention on a specific image, object or thought is guided by a specific fronto-parietal operational module (OM) that serves as an order parameter and determines which particular OM of cortical dynamics (synchronized spatial-temporal pattern of brain activity) should be reinforced at any given moment of time in order to present a particular image, object or thought in the focus of attention.

Finally, it should be noted that the periodic nature of attentional processing is also visible at wider temporal scales: spontaneous eyeblinks, which occur 15–20 times per minute on average, are closely correlated to attentional processing in that they tend to occur at breakpoints of attention, such as the end of a sentence while reading, a pause by the speaker while listening to a speech, and implicit breakpoints while viewing videos. This close correlation has led Nakano et al. (2013, p. 702) to hypothesize that “eyeblinks are actively involved in the process of attentional disengagement during cognitive behavior by momentarily activating the default-mode network while deactivating the dorsal attention network.”

All this evidence points, on the one hand, to the periodic nature of attention and, on the other hand, to the close correlation between the periodicity of attention and brain oscillations. Above all Busch and VanRullen’s (2010) shows two important facts about attention. Firstly, attention has a natural or default periodicity (around 7 Hz): it samples information even when only a single location has to be monitored. Secondly, attention cannot be allocated at any given time but only at specific phases of an oscillatory cycle. It is therefore highly plausible to theorize that attention is the product of, or is underpinned by (one or some of) such brain oscillations.

### WORKING MEMORY

In order to maintain and combine the elements selected and isolated by attention, a specific mechanism is required. This mechanism is working memory.

Working memory is generally considered as a system that helps to simultaneously manipulate information over a short period and update it in memory. More specifically, as highlighted by Unsworth and Engle (2007), working memory is needed to maintain new and novel information in a heightened state of activity, and to correctly discriminate between relevant and irrelevant information with regard to the task to be performed, by preventing the interference of automatic tendencies and routines. In this sense, working memory is “not directly about remembering *per se*, but instead reflects a more general ability to control attention and exert top-down control over cognition” (Broadway and Engle, 2011b, p. 1).

The role of working memory in flexibly and freely combining content elements into new structures is explicitly theorized by Oberauer (2009). According to Oberauer (2009), working memory is a system that is able (among other functions) to build and maintain new structural representations by establishing and holding temporary *bindings* between contents (objects, events, words) and contexts (such as positions in a generic cognitive spatial or coordinate system, or argument variables in structure templates). Neurophysiological studies have started to elucidate this system. Experimental findings using the OA methodology in EEG analysis clearly point to the fact that the binding of sensory feature representations into phenomenal (subjective) “objects,” active encoding, maintenance and retrieval of these mental “objects” during working memory are critically dependent on dynamic millisecond-range synchronization of multiple operations performed by local neuronal assemblies that operate on different temporal (oscillations) scales nested within the same operational hierarchy (Fingelkurts et al., 2010; Monto, 2012). In particular, medium life-span OMs of brain activity (that “cover” certain cortical areas) seem necessary to achieve successful memorization (Fingelkurts et al., 1998, 2003). Indeed, although memory encoding, retention and retrieval often share common regions of the cortex, the operational synchrony of these areas is always unique and presented as a mosaic of nested OMs for each stage of the short-term memory task (Fingelkurts et al., 1998, 2003). When there are too few or too many OMs and their life-span is either too short or too long, then such conditions lead to cessation of efficient memorization.

By supporting arbitrary bindings between virtually any content with any context, working memory enables the compositionality of thought, and the creation of a theoretically unlimited number of different ideas. The binding of contents and contexts allows for the arrangement and representation of objects and events in a spatial and temporal coordinate system, as well as in some other quantitative dimensions such as size, brightness, intelligence, etc.

Recent findings showing that working memory plays a role in the construction of novel future events are provided by Hill and Emery (2013). They started from the observation that, unlike past episodic recall which requires reconstructing the elements of a previously experienced event, future thought depends on a novel recombination of episodic details into a hypothetical event (Addis and Schacter, 2011). This requirement for a novel recombination suggests that future event construction involves additional cognitive and neural processes that are not as involved in autobiographical memory reconstruction. Furthermore, the observed involvement of both prefrontal and hippocampal regions in the construction of future events (Addis et al., 2007), as well as the involvement of the episodic buffer of working memory in prospective mind wandering (Baird et al., 2011) and in recombining semantic personal information (D’Argembeau and Mathy, 2011) and information from multiple modalities (Szpunar et al., 2009), suggests that a combination of executive and memory binding functions may contribute to this novel constructive process. This points to a potential cognitive role for working memory in imagining future episodes, above and beyond the contributions provided by access to the autobiographical database. Using a composite score of working memory capacity (WMC), Hill and Emery (2013) examined the extent to which residual working memory variance contributes to future thought while controlling for autobiographical memory. Subjects had to complete simple and complex measures of working memory and were cued to recall autobiographical memories and imagine future autobiographical events consisting of various levels of specificity (i.e., ranging from generic to increasingly specific and detailed events). They found (1) that the ability to imagine personally relevant events in the future is strongly related to autobiographical memory and (2) that after controlling for autobiographical memory, residual working memory variance *independently* predicts future episodic specificity. That is, when imagining future events, working memory contributes to the construction of a single, coherent, future events depiction.

It is interesting to note that what I propose to be the two main underlying mechanisms of constructive processes – attention and working memory – interact so closely, and seem not to be able to operate without each other, that some scholars have put forward models explicitly including either the former in the latter (Engle, 2002; Oberauer, 2009), or the latter in the former (Knudsen, 2007).

## VARIOUSLY USING ATTENTION AND WORKING MEMORY YIELDS VARIOUS CONSTRUCTION PROCESSES

In order to occur, any construction process requires the availability of some basic elements to be assembled, and a mechanism that allows for the assembly of these basic elements (Benedetti et al., 2010; Fingelkurts et al., 2010, 2013). Regarding the construction processes underpinning the various forms of consciousness, we have seen that the basic elements are provided by attention, and that their assembly is ensured by working memory.

Attention can be variously applied and used: it can be focused internally or externally (Chun et al., 2011); it can be focused at variable levels of size, being set either widely across a display of objects or narrowly to the size of a single object (Jonides, 1983); it can be focused at variable levels of intensity (La Berge, 1983), etc. Likewise, working memory can be variously used to perform arithmetical operations, compare pieces of information, combine items, etc. In some situations it is optimal to maintain as many distinctive items as possible active in working memory, while in some others it is optimal to maintain only one item (Unsworth and Engle, 2007). Some tasks may require a more intensive involvement of the procedural part of working memory as compared to the declarative part, while some others may require the opposite (Oberauer, 2009), etc.

It is precisely the fact that both the selection of basic elements and their assembly can be performed in various ways, that allows various construction processes to be performed, thereby obtaining various forms of consciousness. This parallels any other construction process. Just as a house can be built using bricks rather than stones or wood, so too conscious experiences can be built using current information from the outer world rather than information retrieved from memory. Likewise, just as bricks can be assembled in order to build a corner rather than a wall, so too information retrieved from memory can be combined in order to imagine future events rather than to relive past events.

This paper describes the constructive process that yields the conscious experience of temporality. Therefore, I will specifically focus on which operations attention and working memory perform in order to produce such a conscious experience. However, I will also briefly describe the operations of attention and working memory involved in the construction of some other form of consciousness, so as to provide a set of comparable analyses for future empirical verification.

### THE CONSTRUCTION OF TEMPORAL EVENTS

If one wants to investigate how the construction of temporal events occurs, one must necessarily start from the analysis of its most basic manifestation: duration. The subjective experience of temporality is fundamentally durational in nature, in the sense that duration represents a prerequisite for the development of the other important experiences that are usually conceived as being strictly linked to time, such as the awareness of change, the experience of succession, and the possibility of distinguishing past from present and from future. Some researchers (Gibson, 1975, 1979; Lakoff and Johnson, 1999) maintain the primacy of event detection over duration: according to them, temporal experience would primarily be, and derive from, the awareness of change exhibited by events in the world. However, some facts clearly show that the experience of time is not based on event comparison (for a detailed review, see Evans, 2004). Firstly, we can experience the passage of time whether there has actually been a change in the world-state or not, as evidenced by situations of relative sensory-deprivation (such as windowless, sound-proofed cells) in which subjects are still aware of the passage of time. Secondly, the experience of duration is independent of the nature of external events: the experience of protracted duration can result from both states in which the stimulus array is impoverished and events that, on the contrary, are extremely rich in sense-perceptory terms.

How is then the experience of duration constructed? According to my analysis (Marchetti, 2009a), an event or object assumes a durational dimension when we devote, in an incremental manner, part of our attention to the conscious experience of the event or object. More specifically, the duration of a given event is determined by the cumulative quantity of labor performed by the portion of our attention (*A*_t_) that is kept focused on the conscious experience of the event. Since the event itself is constructed by means of another portion of one’s attention (*A*_e_), duration judgments can be considered equivalent to divided-attention tasks, in which attention must be divided between temporal and non-temporal information processing (Block and Zakay, 2001; Zakay and Block, 2004). The labor performed by *A*_t_ is cumulated thanks to working memory. The cumulative amount of labor performed by *A*_t_ constitutes the basis on which the conscious experience of duration and more in general time-sensation are anchored.

Some alternative explanations of the psychological phenomenon of duration have been put forward, but have various drawbacks. A very well-known alternative is the internal-clock model (Treisman, 1963; Wearden et al., 1998; Wearden, 2001). As an example of internal-clock models, let us consider the “scalar expectancy theory” (SET) proposed by Wearden (2001). The SET model is composed of three parts: a pacemaker-accumulator, a memory system, and a comparison or decision process. To understand how such a model operates, consider the problem of timing the duration of a stimulus *t*_1_ through comparison with the duration of another stimulus, *t*_2_ (whether, for example, they are equal or different in length). Onset of stimulus *t*_1_ causes the pulses, that is, the “ticks” of the inner clock, to flow from the pacemaker to the accumulator. Offset of stimulus causes the interruption of the flow of pulses: the accumulation of pulses by the accumulator is then stopped. The memory system allows duration representations to be stored either in a long-term memory or in a short-term memory. Thanks to the memory system, the duration of the first stimulus *t*_1_ can be stored until after the second one, *t*_2_, has been presented: a comparison between the two stimuli is then possible. Finally, *t*_1_ and *t*_2_ are compared and a response can be delivered.

Internal-clock models can certainly account for some phenomena, such as the differences in judging the duration of auditory stimuli versus visual ones. However, they face various kinds of problems. Generally speaking, internal-clock models seem inadequate to explain the inherent inaccuracy of human duration judgments, that is, the fact that organisms provided with such a precise mechanism as an internal clock very often exhibit inaccurate timing behaviors (Block, 1990). Methodologically speaking, internal-clock models present many drawbacks (Block, 2003), among which the facts that most of the evidence comes from a few relatively simple paradigms (such as the peak procedure and the bisection task), from studies in which animals estimate the duration of a single stimulus or an interval between two stimuli, and from experiments during which no external stimuli are presented internal-clock models cannot readily explain the effects of attention on psychological time; many of the findings that internal-clock models explain are generic, that is, they are not unique to the time dimension. The same findings of internal-clock models could be explained by models composed of very basic modules, such as a perceptual system, without resorting to an additional component such as the pacemaker.

Additionally, internal-clock models face the problem of the individuation of the internal clock (Ornstein, 1969). Apparently, human beings are provided with a number of different mechanisms that could all equally and finely act as internal clocks: heart rate, breathing rate, cellular metabolism, toe-nail growth, alpha rhythm, etc. What are the criteria for judging a given physiological process to be an internal “chronometer”? Why could hair growth or toe-nail growth rather than alpha rhythm not be designated as the internal time keeper? Richelle et al. (1985, p. 90) go so far as to pose the provocative question: “Why not admit that there are as many clocks as there are behaviors exhibiting timing properties?” This admission definitely confirms the uselessness and lack of parsimony of the notion of the internal clock for a general analysis and explanation of time experience.

Finally, the explanation put forward by internal-clock models implies a fallacious circularity (Marchetti, 2010). Merely naming a given process as a “time keeper” or “internal clock” cannot automatically suffice to appoint it as the mechanism responsible for time experience. A counter or a timer, like any clock, can only provide the raw material necessary for counting. But there must be someone who performs the counting. As Vicario argues: “The clock says the hour only when we look at it” (Vicario, 2005, p. 165). It is we who assign the physical mechanism – whether it is a pendulum, the sun, a clock, or something else – the capacity to trace the flowing of our conscious experiences and to estimate their duration. To realize this, just consider the fact that a clock which has stopped or is not working, despite not measuring any actual time, can still be interpreted by an observer as telling the time!

Another possible explanation accounts for time experience in terms of the passage from one conscious state to another: that is, the fact that an event that is being experienced now, becomes no longer present, and passes into the domain of memory. The experience of the passage of time would be produced by the experience of change of conscious state and the phenomenal differences that exist between the various conscious states (the experience of remembering an object is phenomenally different from the experience of actually perceiving it). By taking into account the experiences implied by the change of conscious state, this explanation certainly captures part of the origin of the experience of time. However, it still remains at a very phenomenological and surface level, without investigating the possible neurophysiological mechanisms underpinning the phenomenon. In fact, the experience of the change of conscious state, and of the differences between the various conscious states, can be explained by and reduced to a more basic level: the working of attention and working memory. Actually, in order for one to realize that “something-that-is-present-now” has become “something-that-is-no-longer-present,” one must first isolate the two experiences (the “something-that-is-present-now” and the “something-that-is-no-longer-present”) and then compare them. That is, one must first attentionally focus on them separately, and then keep one of them active in working memory so as to allow for the comparison with the other.

Let us now see what evidence could support my hypothesis about the involvement of attention and working memory in the construction of the experience of duration.

### EVIDENCE OF THE INVOLVEMENT OF ATTENTIONAL AND WORKING MEMORY IN THE EXPERIENCE OF DURATION

Evidence of the attentional basis of the experience of duration is confirmed by a number of empirical findings. Experiments in which subjects are asked to prospectively^1^ judge the duration of the time period in which they had to perform a certain task, reveal that the judged time decreases linearly with the increased processing demands of the non-temporal information, and that experienced duration increases to the extent that subjects can allocate more attentional resources to the flow of time itself (Hicks et al., 1976, 1977; Brown, 1985; Coull et al., 2004). In prospective time judgments, negative high-arousal stimuli, inducing a stronger attentional response, are overestimated compared with positive high-arousal stimuli, inducing a weaker attentional response (Angrilli et al., 1997). Likewise, Tse et al. (2004) found that the engagement of attention by an unexpected event increases the rate of information processing brought to bear on a stimulus, thus inducing an overestimation of the duration of the stimulus.

A confirmation of the role played by attention in constructing temporal experience also comes indirectly from experiments in which subjects are asked to retrospectively^2^ judge the duration of events. Such experiments show that subjects remember a time period as being longer in duration to the extent that there are greater context changes (such as a variation in background stimuli or interoceptive stimuli, the psychological context, the processing context, etc.; Block and Zakay, 2001; Zakay and Block, 2004). Since, as Glicksohn (2001), observes retrospective time estimation entails reperceiving (imaginally) the event, it is conceivable that the retrospective judgment of time is determined (at least in part) by what would have been a prospective judgment of time. Therefore, the phenomena observed in retrospective time estimation can also be ascribed to attention (for a more detailed discussion, see Marchetti, 2010).

Finally, it should be noted that attention has been found to also determine other important aspects of temporal experience not principally related to the experience of duration. For example, the phenomenon known as prior-entry shows that when a person attends to a stimulus, he/she perceives it as having occurred earlier in time than it would if he or she was not attending to it (Shore et al., 2001; Shore and Spence, 2004).

As concerns the role of working memory in the experience of duration, to my knowledge there is no direct evidence as yet of its involvement in the terms that are explained in this work. Broadway and Engle (2011a,b) showed, in a series of temporal reproduction tasks, the close relationship between WMC and duration judgment. Using naturally occurring individual differences in WMC to mimic load manipulations, Broadway and Engle (2011b) found that low-WMC individuals are less sensitive than high-WMC to identifying the longer of two comparison intervals across a range of absolute durations and duration differences. That is, individual differences in WMC predict differences in temporal discrimination. As Broadway and Engle explain, WMC is necessary for performing temporal reproduction tasks because a person would need to encode and maintain access to two distinct representations of elapsed time in an ongoing dynamic manner for comparison and temporal judgment. Therefore, further experiments are needed to verify what my analysis shows, that is, that working memory is necessary to consciously experience duration *per se*, independently of any possible duration judgment.

Several models and empirical findings point specifically to a cumulative build-up mechanism as a possible basis for the experience of duration (for a review, see Wittmann, 2013). It should be noted however that none of them explicitly refer to working memory as the main mechanism responsible for integrating information. According to the dual klepsydra model by Wackermann and Ehm (2006), time duration is represented by the states of inflow-outflow units, which function as leaky integrators. The state of the integrator is a non-linear climbing function of physical time. Craig (2009) theorizes that the anterior insula integrates representations of body states with cognitive and motivational states, creating a series of emotional moments, each of which is a coherent representation of all feelings experienced at that time. The experience of duration would develop from the integration of a series of states over time. Craig’s model suggests that the subjective dilation of time during periods of high emotional salience results from the high rate of salience accumulation, which would rapidly fill up global emotional moments.

From an empirical point of view, neurophysiological findings in primates and humans show that climbing neural activity in several brain regions is related to the experience of duration (Niki and Watanabe, 1979; Lebedev et al., 2008; Mita et al., 2009; Wittmann et al., 2010, 2011; Casini and Vidal, 2011; Merchant et al., 2011). For example, functional fMRI experiments in which subjects have to temporally reproduce acoustic stimuli of various lengths (Wittmann et al., 2010, 2011), show an accumulating pattern of activity within left and right dorsal posterior insula and superior temporal cortex during the encoding phase of the task (with the activity peaking at the end of the interval), and an accumulating activation in the anterior insula, medial frontal and inferior frontal cortex in the reproduction phase of the task (with the activity peaking shortly before the button press indicating the reproduced length by the subject). As Merchant et al. (2013) observe, the ubiquitous increases or decreases in cell discharge rate as a function of time across different timing tasks and brain areas, suggest that ramping activity is a fundamental element of the timing mechanism.

### THE EXPERIENCE OF THE PAST, PRESENT AND FUTURE

As we have seen, we have the capacity to experience the same event as occurring either in the present, past, or future (Nyberg et al., 2010). What construction process makes this possible?

According to my analysis, a temporal event can acquire a past or future dimension if it is placed in a temporal coordinate system having the “present” as its reference point.

As many scholars have highlighted (Revonsuo, 2006; Droege, 2009; Dresp-Langley and Durup, 2012; Fingelkurts and Fingelkurts, 2014), the temporal dimension of “present” is constitutive of conscious experiences^3^. Without such a dimension, there would be no conscious experiences as we currently live them: it is the reference point that allows for the construction of past and future. As Fingelkurts and Fingelkurts (2014) state: “even remembering the past images and planning the future events cannot be performed other than in the present moment and in relation to current state of affairs.” There can be cases of conscious experiences lacking the characteristic of time (as well as of for-me-ness) such as those achieved by trained subjects who practice meditation. However, these cases are very uncommon, and can be attained either in exceptional cases or via extended practice.

Once a temporal event is placed in a temporal coordinate system where the “present” acts a reference point, it can be related to this reference point, and consequently assume either a past or a future property.

How can a temporal coordinate system be constructed? It can be obtained from the most elementary and primitive experience of time, that is, duration. As we saw, the experience of duration is based on the *cumulative* quantity of labor performed by the portion of attention (*A*_t_) that is kept focused on the conscious experience of the event. Being cumulative, the quantity of labor performed by *A*_t_ can only increase. This makes it possible to arrange events in a univocal and irreversible way, which is precisely the condition necessary to construct a temporal coordinate system made of “past,” “present,” and “future.” Generally speaking, if we consider for example that a given event X can be associated with a certain amount of labor performed by *A*_t_, an event Y that is associated with a higher amount of labor performed by *A*_t_ appears to us to happen “after” X, whereas an event Z that is associated with a lower amount of labor performed by *A*_t_ appears to happen “before” X. That is, once X, Y, and Z are assigned a specific location in a cognitive coordinate system characterized by one-dimensionality and irreversibility, they are ordered according to the temporal dimension. More specifically, if event X occurs in the “present,” Z will be experienced as occurring in the “past” and Y as occurring in the “future.”

The existence of past and future makes it possible to construct the conscious experiences of remembering past events and imagining future events. As suggested by Ceccato and Zonta’s (1980) work, and more specifically by phenomenological analysis (see Thompson, 2008), the subjective experience of remembering an event derives from adding the temporal dimension of past to the event. In remembering, one lives experiences as having occurred in the “past” and not as occurring now. In a similar way, the subjective experience of imagining a future event derives from adding the temporal dimension of future to the event. The operation of adding a (past or future) temporal dimension to an event is performed thanks to working memory, which binds the event to a position in the temporal coordinate system (Oberauer, 2009).

Empirical evidence supporting this analysis is still partial and indirect, and a specific investigation must be performed in order to validate the analysis. As we have seen, Hill and Emery’s (2013) work confirms the role played by working memory in mental time travel, specifically when imagining future events. The close link between attention and the conscious experience of reliving past events was reviewed by De Brigard (2012). Behavioral studies using divided attention paradigms show that when internal attention (Chun et al., 2011) to material-congruent deeply encoded information is disrupted during retrieval, recollection is significantly impaired (Fernandes and Moscovitch, 2000; Hicks and Marsh, 2000). Likewise, neuropsychological studies show that under free-recall conditions, patients with parietal cortex damage, which usually impairs attention to external stimuli, tend to retrieve less episodic perceptual details and lower levels of vividness in their recollections from their autobiographical memories relative to both cued-recall and healthy controls (Berryhill et al., 2007). Davidson et al. (2008) also show that patients with parietal lesions produce a reduced number of “remember” responses, which are associated with increased subjective experience of recollection, relative to both “know” responses and controls.

To summarize the analysis I have put forward: the conscious experience of duration is produced by two (non-conscious) mechanisms: attention and working memory. The conscious experiences of past, present and future are in turn built on the conscious experience of duration. By adding the temporal dimensions of past and future to an event, it is possible to subjectively experience that event as remembered or occurring in the future, respectively.

This kind of explanation of temporal experience does not rely on mechanisms purposefully designed to process time (such as an “internal-clock”), but rather on mechanisms (attention and working memory) that have other, more basic and general-purpose functions, not necessarily related to the encoding of duration and time. As such, the circularity implied in many other explanations of temporal experience (Marchetti, 2009a) is avoided.

### HOW THE CONSTRUCTION PROCESS WORKS: A COMPARISON WITH SOME OTHER FORMS OF CONSCIOUSNESS

As we have seen, the attentional selection of basic elements and their assembly by means of working memory can be performed in various ways. This allows various construction processes to be performed, thereby obtaining various forms of consciousness. Here I will briefly show how variously using attention and working memory yields different forms of consciousness (see **Table 1** for an overview). In this way, I intend to provide an initial set of comparable analyses that can be used to empirically verify my analyses.

**Table 1:** The different involvement of attention and working memory in some forms of consciousness.

Form of consciousness	Form of attention	Focus of attention	Activity performed by working memory (WM)
Duration	Internal attention	Conscious experience of the event whose duration is judged	Integration of attentional states
Episodic memory	Internal attention	The event located in the past	Supporting the arrangement of the event in a temporal coordinate system
Episodic future thought	Internal attention	The event located in the future	Supporting the arrangement of the event in a temporal coordinate system
Space	Internal and external attention	Products of the activity performed by proprioceptors, vestibular system and sense-organs	Integration of attentional states
Language	Internal attention, shared attention, etc.	Semantic memory, short term- memory, interlocutors	Supporting relational units that variously combine semantic elements. The level of WM involvement varies according to sentence structure and length
Thought	Internal attention	Representational systems, frames, specific domain knowledge	Supporting (conscious and unconscious) operators that variously combine elements of various nature. The level of WM involvement is high

#### The conscious experience of space

The conscious experience of space is primarily based on bodily movements (Berthoz, 2000; Morris, 2004). However, bodily movements, albeit necessary, are not sufficient (Marchetti, 2009b). The conscious experience of space also requires that: (a) attention is internally applied to prorioceptors and the vestibular system in order to isolate and bring to consciousness the single perceptions entailed by movement. External attention is also required to build some conscious experiences of space (Berthoz, 2000); (b) working memory assemblies these single perceptions, by keeping them present in an incremental way. It is this latter operation that allows for the construction of a “sequence” or “succession” of perceptions, which is the basis for the formation of two-dimensional constructs, such as “path,” “line,” and “distance.”

The need for working memory (in addition to bodily movement) in the construction of the conscious experience of space is evident when comparing the different conscious experiences of “movement” and “line” (or “path”) we have when performing the same act. For example, move your index finger slowly. Now, look at the tip of the finger while the finger moves, and consider it as a moving object. Next, repeat the movement and consider the path or line drawn by the tip of the finger. You will notice that in the former case you will simply follow the tip of the finger, maybe anticipating its direction, but without keeping track of the positions previously occupied by it; on the contrary, in the latter case you will follow the tip of the finger by constantly keeping track of the positions it occupied, moment after moment, since it started moving.

#### Language and conscious thinking

Language allows us to variously and, theoretically, endlessly combine meanings. This combinatorial power is made possible by relational units (such as conjunctions and prepositions: Sapir, 1921; Ceccato, 1972; Ceccato and Zonta, 1980; Benedetti, 2006, 2009, 2011), which, tying together two or more semantic elements (simple words, other relational units, complex thoughts, etc.), allow for the construction of correlational networks (or minimal unit of linguistic thought).

As shown by Benedetti’s (2006), analysis the production of correlational networks is made possible by working memory and procedural memory. These two forms of memory, albeit necessary, are not sufficient. Theoretically, correlational networks can be infinite. However, the well-known limits of working memory constrain this possibility. In fact, sentences have a limited length and are separated by semicolons, full stops, pauses, etc. At full stops, working memory stops being loaded, and what has been present in it up to that moment, must be in some way stored in a summarized form in a short-term memory. Pronouns have the function of reloading into working memory what has been stored in short-term memory.

It should be noted that, compared and contrary to the experiences of time and space, language does not specifically require that working memory cumulates attentional states. In language, working memory has the primary and most general function of supporting relational units in (variously) combining semantic elements. Moreover, language specifically needs the involvement of semantic memory, which is only rarely, or indirectly, involved when constructing temporal and spatial experiences. Additionally, language deeply requires other forms of attention, such as shared attention (Tomasello, 1999, 2004; Oakley, 2009).

Language and the kind of thought it entails (linguistic or correlational thought: Ceccato and Zonta, 1980; Benedetti, 2011) can be considered a specific subset of conscious thinking (Marchetti, 2010). In many cases, we have dynamic and evolving visuo-spatial thought (such as when we think, for example, about a flower that opens), or forms of thoughts involving other senses. Moreover, most of the times, thoughts do not just combine elements, but produce results, such as when a solution suddenly pops into one’s mind after having searched for it for a while. Generally speaking, conscious thinking requires operators other than just the relational units involved by language: these operators should allow, for example, for the transformation of the object of thought, or the production of new conscious experiences from earlier ones, or the comparison of elements.

Therefore, conscious thinking, compared to language, sometimes requires a deeper involvement of working memory (such as when evolving representations are produced), and the presence of dedicated (and usually unconscious) frames, representational and operational systems that are not strictly required by language (such as those that allow one to draw inferences, or make decisions).

A final consideration should be made about the empirical verification of the analyses I have put forward here and elsewhere (Marchetti, 1997, 2010). Generally speaking, it should be noted that these analyses are particularly suited to be verified by an empirical approach centered on the notion of operation and its combinatorial power. In fact, my analyses describe in a sufficiently detailed way what operations (that is, how basic elements are assembled and combined) must be performed in order to obtain certain forms of consciousness. As already shown in another paper (Benedetti et al., 2010), the Fingelkurts brothers’ OA (Fingelkurts and Fingelkurts, 2001, 2005; Fingelkurts et al., 2009, 2010, 2012, 2013) offers such an empirical approach. According to OA, simple cognitive operations that present some partial aspect of an object/scene/concept or thought are presented in the brain by local 3D-fields produced by discrete and transient neuronal assemblies, which can be recorded by an EEG. More complex operations that constitute the whole object/scene or thought are brought into existence by joint (synchronized) simple operations in the form of coupled 3D-fields – so called OMs of varied complexity. OA does not put forward specific analyses in operational terms of phenomenological contents and forms. However, because of the hierarchical organization implied by its theoretical framework, OA is very suited to verify precisely this kind of analysis.

## CONCLUSION

This article is an attempt to demonstrate that some of the most important forms of consciousness – episodic memory, episodic future thought, perception, language and conscious thinking – are based on an active constructive process. Despite the fact that we experience the world surrounding us as a continuous, seamless flow of information, many psychological and neurophysiological observations reveal that information is actually extracted and processed in distinct moments, similar to the snapshots of a camera.

For most of the forms of consciousness to occur, a construction combining the various moments or snapshots is required. The main plausible mechanisms implied in this construction process are attention and working memory. Attention allows for the selection of the basic elements to be assembled. Empirical evidence shows that attention works on a period basis (around 7 Hz): it samples information even when only a single location has to be monitored. Working memory represents the mechanism that allows for the assembly of the basic elements selected by attention, by establishing and holding temporary *bindings* between contents and contexts.

Both the selection of basic elements and their assembly can be performed in various ways, thus allowing various construction processes to be performed, thereby obtaining various forms of consciousness.

Temporal experience is based on a specific kind of working of attention and combination of attentional moments. Namely, the duration of a given event is determined by the cumulative quantity of labor performed by the portion of our attention (*A*_t_) that is kept focused on the conscious experience of the event. The labor performed by *A*_t_ is cumulated thanks to working memory. The experience of duration provides the basis for the construction of the conscious experiences of past, present and future. By adding the temporal dimensions of past and future to an event, it is possible to subjectively experience that event as remembered or occurring in the future.

## Conflict of Interest Statement

The author declares that the research was conducted in the absence of any commercial or financial relationships that could be construed as a potential conflict of interest.

## Footnotes

1 That is subjects are alerted in advance that time judgments will be required.

2 That is subjects do not know in advance that they will later be asked to judge the duration of a time period.

3 The dimension of “present” is not the only constitutive feature of consciousness. Some other dimensions have also been identified, such as the dimensions of “here” and “for-me-ness,” that is, the fact that of all of our experiences are characterized implicitly by a quality of mineness: see Revonsuo (2006) and Gallagher and Zahavi (2008).

## References

[B1] Addis D. R. Schacter D. L. (2011). The hippocampus and imagining the future: where do we stand? *Front. Hum. Neurosci.* 5:173 10.3389/fnhum.2011.00173 PMC3251274 22291625

[B2] Addis D. R. Wong A. T. Schacter D. L. (2007). Remembering the past and imagining the future: common and distinct neural substrates during event construction and elaboration. *Neuropsychologia* 45 1363–1377 10.1016/j.neuropsychologia.2006.10.016 17126370 PMC1894691

[B3] Angrilli A. Cherubini P. Pavese A. Manfredini S. (1997). The influence of affective factors on time perception. *Percept. Psychophys.* 59 972–982 10.3758/BF03205512 9270369

[B4] Baird B. Smallwood J. Schooler J. W. (2011). Back to the future: autobiographical planning and the functionality of mind-wandering. *Conscious. Cogn.* 20 1604–1611 10.1016/j.concog.2011.08.007 21917482

[B5] Baumeister R. F. Masicampo E. J. (2010). Conscious thought is for facilitating social and cultural interactions: how mental simulations serve the animal-culture interface. *Psychol. Rev.* 117 945–971 10.1037/a0019393 20658859

[B6] Benedetti G. (2006). Operational noology as a new methodology for the study of thought and language: theoretical aspects and possible practical applications. *Cogn. Process.* 7 217–243 10.1007/s10339-006-0145-8 16897066

[B7] Benedetti G. (2009). The meaning of the basic elements of language in terms of cognitive operations: operational semantics. *Adv. Stud. Biol.* 1 255–305

[B8] Benedetti G. (2011). *An Enigma in Language. The Meaning of the Fundamental Linguistic Elements. A Possible Explanation in terms of Cognitive Functions: Operational Semantics*. New York: Nova Science Publishers.

[B9] Benedetti G. Marchetti G. Fingelkurts A. A. Fingelkurts A. A. (2010). Mind operational semantics and brain operational architectonics: a putative correspondence. *Open Neuroimag. J.* 4 53–69 10.2174/1874440001004020053 21113277 PMC2948146

[B10] Berryhill M. E. Phuong L. Picasso L. Cabeza R. Olson I. R. (2007). Parietal lobe and episodic memory: bilateral damage causes impaired free recall of autobiographical memory. *J. Neurosci.* 27 14415–14423 10.1523/JNEUROSCI.4163-07.2007 18160649 PMC6673454

[B11] Berthoz A. (2000). *The Brain’s Sense of Movement*. Cambridge: Harvard University Press

[B12] Blais C. Arguin M. Gosselin F. (2013). Human visual processing oscillates: evidence from a classification image technique. *Cognition* 128 353–362 10.1016/j.cognition.2013.04.009 23764998

[B13] Block R. A. (1990). “Model of psychological time,” in *Cognitive Models of Psychological Time*, ed. R. A. Block (Hillsdale, NJ: Lawrence Erlbaum Associates).

[B14] Block R. A. (2003). “Psychological timing without a timer: the roles of attention and memory,” in *Time and Mind II: Information Processing Perspectives*, ed. H. Helfrich (Göttingen: Hogrefe & Huber Publishers).

[B15] Block R. A. Zakay D. (2001). “Retrospective and prospective timing: memory, attention, and consciousness,” in *Time and Memory: Issues in Philosophy and Psychology* eds Hoerl C. McCormack T. (Oxford: Oxford University Press), 59–76

[B16] Bor D. Seth A. K. (2012). Consciousness and the prefrontal parietal network: insights from attention, working memory, and chunking. *Front. Psychol.* 3:63 10.3389/fpsyg.2012.00063 PMC3298966 22416238

[B17] Boynton G. M. (2005). Attention and visual perception. *Curr. Opin. Neurobiol.* 15 465–469 10.1016/j.conb.2005.06.009 16023853

[B18] Broadway J. M. Engle R. W. (2011a). Lapsed attention to elapsed time? Individual differences in working memory capacity and temporal reproduction. *Acta Psychol.* 137 115–126 10.1016/j.actpsy.2011.03.008 21470583

[B19] Broadway J. M. Engle R. W. (2011b). Individual differences in working memory capacity and temporal discrimination. *PLoS ONE* 6:e25422 10.1371/journal.pone.0025422 PMC3189201 22003391

[B20] Brown S. W. (1985). Time perception and attention: the effects of prospective versus retrospective paradigms and task demands on perceived duration. *Percept. Psychophys.* 38 115–124 10.3758/BF03198848 4088803

[B21] Busch N. A. Dubois J. VanRullen R. (2009). The phase of ongoing EEG oscillations predicts visual perception. *J. Neurosci.* 29 7869–7876 10.1523/JNEUROSCI.0113-09.2009 19535598 PMC6665641

[B22] Busch N. A. VanRullen R. (2010). Spontaneous EEG oscillations reveal periodic sampling of visual attention. *Proc. Natl. Acad. Sci. U.S.A.* 107 16048–16053 10.1073/pnas.1004801107 20805482 PMC2941320

[B23] Buschman T. J. Miller E. K. (2010). Shifting the spotlight of attention: evidence for discrete computations in cognition. *Front. Hum. Neurosci.* 4:194 10.3389/fnhum.2010.00194 PMC2990535 21119775

[B24] Carrasco M. (2011). Visual attention: the past 25 years. *Vision Res.* 51 1484–1525 10.1016/j.visres.2011.04.012 21549742 PMC3390154

[B25] Casini L. Vidal F. (2011). The SMAs: neural substrate of the temporal accumulator? *Front. Integr. Neurosci.* 5:35 10.3389/fnint.2011.00035 PMC3154296 21886611

[B26] Ceccato S. (1972). *La Mente Vista da un Cibernetico*. Torino: Eri

[B27] Ceccato S. Zonta B. (1980). *Linguaggio Consapevolezza Pensiero*. Milano: Feltrinelli

[B28] Chafe W. (1994). *Discourse, Consciousness, and Time. The Flow and Displacement of Conscious Experience in Speaking and Writing*. Chicago: The University of Chicago Press

[B29] Chun M. M. Golomb J. D. Turk-Browne N. B. (2011). A taxonomy of external and internal attention. *Annu. Rev. Psychol.* 62 73–101 10.1146/annurev.psych.093008.100427 19575619

[B30] Conway C. M. Christiansen M. H. (2001). Sequential learning in non-human primates. *Trends Cogn. Sci.* 5 539–546 10.1016/S1364-6613(00)01800-3 11728912

[B31] Coull J. T. Vidal F. Nazarian B. Macar F. (2004). Functional anatomy of the attentional modulation of time estimation. *Science* 303 1506–1508 10.1126/science.1091573 15001776

[B32] Craig A. D. (2009). How do you feel – now? The anterior insula and human awareness. *Nat. Rev. Neurosci.* 10 59–70 10.1038/nrn2555 19096369

[B33] Crovitz H. F. Schiffman H. (1974). Frequency of episodic memories as a function of their age. *Bull. Psychon. Soc.* 4 517–518 10.3758/BF03334277

[B34] D’Argembeau A. Mathy A. (2011). Tracking the construction of episodic future thoughts. *J. Exp. Psychol. Gen.* 140 258–271 10.1037/a0022581 21401291

[B35] D’Argembeau A. Van der Linden M. (2004). Phenomenal characteristics associated with projecting oneself back into the past and forward into the future: influence of valence and temporal distance. *Conscious. Cogn.* 13 844–858 10.1016/j.concog.2004.07.007 15522635

[B36] Davidson P. S. Anaki D. Ciaramelli E. Cohn M. Kim A. S. Murphy K. J. (2008). Does lateral parietal cortex support episodic memory? Evidence from focal lesion patients. *Neuropsychologia* 46 1743–1755 10.1016/j.neuropsychologia.2008.01.011 18313699 PMC2806230

[B37] De Brigard F. (2012). The role of attention in conscious recollection. *Front. Psychol.* 3:29 10.3389/fpsyg.2012.00029 PMC3276975 22363305

[B38] Dehaene S. (1993). Temporal oscillations in human perception. *Psychol. Sci.* 4 264–270 10.1111/j.1467-9280.1993.tb00273.x

[B39] Doesburg S. M. Green J. J. McDonald J. J. Ward L. M. (2009). Rhythms of consciousness: binocular rivalry reveals large-scale oscillatory network dynamics mediating visual perception. *PLoS ONE* 4:e6142 10.1371/journal.pone.0006142 PMC2702101 19582165

[B40] Doesburg S. M. Roggeveen A. B. Kitajo K. Ward L. M. (2008). Large-scale gamma-band phase synchronization and selective attention. *Cereb. Cortex* 18 386–396 10.1093/cercor/bhm073 17556771

[B41] Dresp-Langley B. Durup J. (2012). Does consciousness exist independently of present time and present time independently of consciousness? *Open J. Philos.* 2 45–49 10.4236/ojpp.2012.21007

[B42] Drewes J. VanRullen R. (2011). This is the rhythm of your eyes: the phase of ongoing electroencephalogram oscillations modulates saccadic reaction time. *J. Neurosci.* 31 4698–4708 10.1523/JNEUROSCI.4795-10.2011 21430168 PMC6622921

[B43] Droege P. (2009). Now or never: how consciousness represents time. *Conscious. Cogn.* 18 78–90 10.1016/j.concog.2008.10.006 19041266

[B44] Duncan J. (2013). The structure of cognition: attentional episodes in mind and brain. *Neuron* 80 35–50 10.1016/j.neuron.2013.09.015 24094101 PMC3791406

[B45] Engle R. W. (2002). Working memory capacity as executive attention. *Curr. Dir. Psychol. Sci.* 11 19–23 10.1111/1467-8721.00160

[B46] Evans V. (2004). *The Structure of Time. Language, Meaning and Temporal Cognition*. Amsterdam-Philadelphia: John Benjamins Publishing Company 10.1075/hcp.12

[B47] Fernandes M. A. Moscovitch M. (2000). Divided attention and memory: evidence of substantial interference effects at retrieval and encoding. *J. Exp. Psychol. Gen.* 129 155–176 10.1037/0096-3445.129.2.155 10868332

[B48] Fingelkurts A. A. Fingelkurts A. A. (2001). Operational architectonics of the human brain biopotential field: towards solving the mind-brain problem. *Brain Mind* 2 261–296 10.1023/A:101442782273

[B49] Fingelkurts A. A. Fingelkurts A. A. (2005). “Mapping of the brain operational architectonics Chapter 2” in *Focus on Brain Mapping Research*, ed. F. J. Chen (New York: Nova Science Publishers, Inc.), 59–98

[B50] Fingelkurts A. A. Fingelkurts A. A. (2014). Present moment, past, and future: mental kaleidoscope. *Front. Psychol.* 5:395 10.3389/fpsyg.2014.00395 PMC4013454 24822050

[B51] Fingelkurts A. A. Fingelkurts A. A. (in press) “Attentional state: from automatic detection to willful focused concentration,” in *Attention and Meaning. The Attentional Basis of Meaning* eds Marchetti G. Benedetti G. Alharbi A. (New York: Nova Science Publishers, Inc.).

[B52] Fingelkurts A. A. Fingelkurts A. A. Ivashko R. M. Kaplan A. Y. (1998). EEG analysis of operational synchrony between human brain cortical areas during memory task performance. *Vestn. Moskovsk. Univer.* 1 3–11

[B53] Fingelkurts A. A. Fingelkurts A. A. Krause C. M. Kaplan A. Y. Borisov S. V. Sams M. (2003). Structural (operational) synchrony of EEG alpha activity during an auditory memory task. *Neuroimage* 20 529–542 10.1016/S1053-8119(03)00305-7 14527613

[B54] Fingelkurts A. A. Fingelkurts A. A. Neves C. F. H. (2009). Phenomenological architecture of a mind and operational architectonics of the brain: the unified metastable continuum. *J. New Math. Nat. Comput.* 5 221–244 10.1142/S1793005709001258

[B55] Fingelkurts A. A. Fingelkurts A. A. Neves C. F. H. (2010). Natural world physical, brain operational, and mind phenomenal space–time. *Phys. Life Rev.* 7 195–249 10.1016/j.plrev.2010.04.001 20417160

[B56] Fingelkurts A. A. Fingelkurts A. A. Neves C. F. H. (2012). “Machine” consciousness and “artificial” thought: an operational architectonics model guided approach. *Brain Res.* 1428 80–92 10.1016/j.brainres.2010.11.079 21130079

[B57] Fingelkurts A. A. Fingelkurts A. A. Neves C. F. H. (2013). Consciousness as a phenomenon in the operational architectonics of brain organization: criticality and self-organization considerations. *Chaos Solit. Fract.* 55 13–31 10.1016/j.chaos.2013.02.007

[B58] Gallagher S. Zahavi D. (2008). *The Phenomenological Mind. An Introduction to Philosophy of Mind and Cognitive Science*. New York: Routledge

[B59] Galton F. (1880). Statistics of mental imagery. *Mind* 5 301–318 10.1093/mind/os-V.19.301

[B60] Gibson J. (1975). “Events are perceivable but time is not,” in *The Study of Time II* eds Fraser J. T. Lawrence N. (New York: Springer-Verlag).

[B61] Gibson J. (1979). *The Ecological Approach to Visual Perception*. Hillsdale, NJ: Lawrence Erlbaum

[B62] Glicksohn J. (2001). Temporal cognition and the phenomenology of time: a multiplicative function for apparent duration. *Conscious. Cogn.* 10 1–25 10.1006/ccog.2000.0468 11273623

[B63] Hanslmayr S. Gross J. Klimesch W. Shapiro K. L. (2011). The role of alpha oscillations in temporal attention. *Brain Res. Rev.* 67 331–343 10.1016/j.brainresrev.2011.04.002 21592583

[B64] Hicks R. E. Miller G. W. Gaes G. Bierman K. (1977). Concurrent processing demands and the experience of time-in-passing. *Am. J. Psychol.* 90 431–446 10.2307/1421874

[B65] Hicks R. E. Miller G. W. Kinsbourne M. (1976). Prospective and retrospective judgments of time as a function of amount of information processed. *Am. J. Psychol.* 90 431–446 10.2307/1421874 1020767

[B66] Hicks J. L. Marsh R. L. (2000). Toward specifying the attentional demands of recognition memory. *J. Exp. Psychol. Learn. Mem. Cogn.* 26 1483–1498 10.1037/0278-7393.26.6.1483 11185778

[B67] Hill P. F. Emery L. J. (2013). Episodic future thought: contributions from working memory. *Conscious. Cogn.* 22 677–683 10.1016/j.concog.2013.04.002 23681207

[B68] Hirsh I. J. Sherrick C. (1961). Perceived order in different sense modalities. *J. Exp. Psychol.* 62 423–432 10.1037/h0045283 13907740

[B69] Jones M. R. Moynihan H. MacKenzie N. Puente J. (2002). Temporal aspects of stimulus driven attending in dynamic arrays. *Psychol. Sci.* 13 313–319 10.1111/1467-9280.00458 12137133

[B70] Jonides J. (1983). Further toward a model of the mind’s eye’s movement. *Bull. Psychon. Soc.* 21 247–250 10.3758/BF03334699

[B71] Kane M. J. Poole B. J. Tuholski S. W. Engle R. W. (2006). Working memory capacity and the top-down control of visual search: exploring the boundaries of “executive attention”. *J. Exp. Psychol. Learn. Mem. Cogn.* 32 749 10.1037/0278-7393.32.4.749 16822145

[B72] Knudsen E. I. (2007). Fundamental components of attention. *Annu. Rev. Neurosci.* 30 57–78 10.1146/annurev.neuro.30.051606.094256 17417935

[B73] Kranczioch C. Debener S. Maye A. Engel A. K. (2007). Temporal dynamics of access to consciousness in the attentional blink. *Neuroimage* 37 947–955 10.1016/j.neuroimage.2007.05.044 17629501

[B74] Kwan D. Craver C. F. Green L. Myerson J. Boyer P. Rosenbaum R. S. (2012). Future decision-making without episodic mental time travel. *Hippocampus* 22 1215–1219 10.1002/hipo.20981 21997930 PMC3262098

[B75] La Berge D. (1983). The spatial extent of attention to letters and words. *J. Exp. Psychol. Hum. Percept. Perform.* 9 371–379 10.1037/0096-1523.9.3.371 6223977

[B76] Lakoff G. Johnson M. (1999). *Philosophy in the Flesh: The Embodied Mind and its Challenge to Western Thought*. New York: Basic Books

[B77] Landau A. N. Fries P. (2012). Attention samples stimuli rhythmically. *Curr. Biol.* 22 , 1000–1004 10.1016/j.cub.2012.03.054 22633805

[B78] Latour P. L. (1967). Evidence of internal clocks in the human operator. *Acta Psychol.* 27 341–348 10.1016/0001-6918(67)90078-9 6062228

[B79] Lebedev M. A. O’doherty J. E. Nicolelis M. A. (2008). Decoding of temporal intervals from cortical ensemble activity. *J. Neurophysiol.* 99 166–186 10.1152/jn.00734.2007 18003881

[B80] Marchetti G. (1997). *La Macchina Estetica. Il Percorso Operativo Nella Costruzione Dell’atteggiamento Estetico*. Milano: Franco Angeli

[B81] Marchetti G. (2009a). Studies on time: a proposal on how to get out of circularity. *Cogn. Process.* 10 7–40 10.1007/s10339-008-0215-1 18504631

[B82] Marchetti G. (2009b). *Commentary on David Morris’. “The Sense of Space”*. Available at: http://www.mind-consciousness-language.com/Commentary Morris.pdf, 1–22

[B83] Marchetti G. (2010). *Consciousness, Attention, and Meaning*. New York: Nova Science Publishers

[B84] Marchetti G. (2012). Against the view that consciousness and attention are fully dissociable. *Front. Psychol.* 3:36 10.3389/fpsyg.2012.00036 PMC3279725 22363307

[B85] Mathewson K. E. Gratton G. Fabiani M. Beck D. M. Ro T. (2009). To see or not to see: prestimulus α phase predicts visual awareness. *J. Neurosci.* 29 2725–2732 10.1523/JNEUROSCI.3963-08.2009 19261866 PMC2724892

[B86] Merchant H. Harrington D. L. Meck W. H. (2013). Neural basis of the perception and estimation of time. *Annu. Rev. Neurosci.* 36 313–336 10.1146/annurev-neuro-062012-170349 23725000

[B87] Merchant H. Zarco W. Pérez O. Prado L. Bartolo R. (2011). Measuring time with different neural chronometers during a synchronization-continuation task. *Proc. Natl. Acad. Sci. U.S.A.* 108 19784–19789 10.1073/pnas.1112933108 22106292 PMC3241773

[B88] Mita A. Mushiake H. Shima K. Matsuzaka Y. Tanji J. (2009). Interval time coding by neurons in the presupplementary and supplementary motor areas. *Nat. Neurosci.* 12 502–507 10.1038/nn.2272 19252498

[B89] Monto S. (2012). Nested synchrony – a novel cross-scale interaction among neuronal oscillations. *Front. Physiol.* 3:384 10.3389/fphys.2012.00384 PMC3458414 23055985

[B90] Morris D. (2004). *The Sense of Space*. Albany, NY: SUNY

[B91] Nakano T. Kato M. Morito Y. Itoi S. Kitazawa S. (2013). Blink-related momentary activation of the default mode network while viewing videos. *Proc. Natl. Acad. Sci. U.S.A.* 110 , 702–706 10.1073/pnas.1214804110 23267078 PMC3545766

[B92] Niki H. Watanabe M. (1979). Prefrontal and cingulate unit activity during timing behavior in the monkey. *Brain Res.* 171 213–224 10.1016/0006-8993(79)90328-7 111772

[B93] Nyberg L. Kim A. S. Habib R. Levine B. Tulving E. (2010). Consciousness of subjective time in the brain. *Proc. Natl. Acad. Sci. U.S.A.* 107 22356–22359 10.1073/pnas.1016823108 21135219 PMC3009795

[B94] Oakley T. (2009). *From Attention to Meaning: Explorations is Semiotics, Linguistics, and Rhetoric*. *European Semiotics Series,* Vol. 8. Berlin: Peter Lang Verlag

[B95] Oberauer K. (2009). Design for a working memory. *Psychol. Learn. Motiv.* 51 45–100 10.1016/S0079-7421(09)51002-X

[B96] Ornstein R. E. (1969). *On the Experience of Time*. Middlesex, England: Penguin Books

[B97] Peters J. Büchel C. (2010). Episodic future thinking reduces reward delay discounting through an enhancement of prefrontal-mediotemporal interactions. *Neuron* 66 138–148 10.1016/j.neuron.2010.03.026 20399735

[B98] Pöppel E. (1997). A hierarchical model of temporal perception. *Trends Cogn. Sci.* 1 56–61 10.1016/S1364-6613(97)01008-5 21223864

[B99] Pöppel E. (2004). Lost in time: a historical frame, elementary processing units and the 3-second window. *Acta Neurobiol. Exp.* 64 295–302 10.55782/ane-2004-1514 15283473

[B100] Revonsuo A. (2006). *Inner Presence: Consciousness as a Biological Phenomenon*. Cambridge, MA: MIT Press

[B101] Richelle M. Lejeune H. Perikel J.-J. Fery P. (1985). “From biotemporality to nootemporality: toward an integrative and comparative view of time in behavior,” in *Time, Mind, and Behavior* eds Michon J. A. Jackson J. L. (Berlin: Springer-Verlag).

[B102] Romei V. Gross J. Thut G. (2010). On the role of prestimulus alpha rhythms over occipito-parietal areas in visual input regulation: correlation or causation? *J. Neurosci.* 30 8692–8697 10.1523/JNEUROSCI.0160-10.2010 20573914 PMC6634639

[B103] Rosenfield I. (1988). *The Invention of Memory: A New View of the Brain*. New York: Basic Books

[B104] Sapir E. (1921). *Language: An Introduction to the Study of Speech*. New York: Harcourt, Brace & Co.

[B105] Schacter D. L. (1999). The seven sins of memory: insights from psychology and cognitive neuroscience. *Am. Psychol.* 54 182 10.1037/0003-066X.54.3.182 10199218

[B106] Schacter D. L. Addis D. R. Hassabis D. Martin V. C. Spreng R. N. Szpunar K. K. (2012). The future of memory: remembering, imagining, and the brain. *Neuron* 76 677–694 10.1016/j.neuron.2012.11.001 23177955 PMC3815616

[B107] Shallice T. (1964). The detection of change and the perceptual moment hypothesis. *Br. J. Stat. Psychol.* 17 113–135 10.1111/j.2044-8317.1964.tb00254.x

[B108] Shore D. I. Spence C. (2004). “Prior entry,” in *Neurobiology of Attention*, eds Itti L. Rees G. Tsotsos J. (Elsevier: North Holland).

[B109] Shore D. I. Spence C. Klein R. M. (2001). Visual prior entry. *Psychol. Sci.* 12 205–212 10.1111/1467-9280.00337 11437302

[B110] Simons D. J. Levin D. T. (1997). Change blindness. *Trends Cogn. Sci.* 1 261–267 10.1016/S1364-6613(97)01080-2 21223921

[B111] Simpson W. A. Shahani U. Manahilov V. (2005). Illusory percepts of moving patterns due to discrete temporal sampling. *Neurosci. Lett.* 375 23–27 10.1016/j.neulet.2004.10.059 15664116

[B112] Suddendorf T. Addis D. R. Corballis M. C. (2009). Mental time travel and the shaping of the human mind. *Philos. Trans. R. Soc. B Biol. Sci.* 364 1317–1324 10.1098/rstb.2008.0301 PMC2666704 19528013

[B113] Suddendorf T. Corballis M. C. (1997). Mental time travel and the evolution of the human mind. *Genet. Soc. Gen. Psychol. Monogr.* 123 133–167 9204544

[B114] Szpunar K. K. (2010). Episodic future thought an emerging concept. *Perspect. Psychol. Sci.* 5 142–162 10.1177/1745691610362350 26162121

[B115] Szpunar K. K. Chan J. C. McDermott K. B. (2009). Contextual processing in episodic future thought. *Cereb. Cortex* 19 1539–1548 10.1093/cercor/bhn191 18980949

[B116] Thompson E. (2008). Representationalism and the phenomenology of mental imagery. *Synthese* 160 397–415 10.1007/s11229-006-9086-0

[B117] Tomasello M. (1999). *The Cultural Origins of Human Cognition*. Cambridge, MA: Harvard University Press

[B118] Tomasello M. (2004). *Constructing a Language: A Usage-Based Theory of Language Acquisition*. Cambridge: Harvard University Press

[B119] Treisman M. (1963). Temporal discrimination and the indifference interval: implications for a model of the ‘internal clock’. *Psychol. Monogr.* 77 1–31 10.1037/h0093864 5877542

[B120] Treue S. (2001). Neural correlates of attention in primate visual cortex. *Trends Neurosci.* 24 295–300 10.1016/S0166-2236(00)01814-2 11311383

[B121] Tse P. U. Intriligator J. Rivest J. Cavanagh P. (2004). Attention and the subjective expansion of time. *Percept. Psychophys.* 66 1171–1189 10.3758/BF03196844 15751474

[B122] Tulving E. (1972). “Episodic and semantic memory,” in *Organization of Memory* eds Tulving E. Donaldson W. (New York: Academic Press), 381–402

[B123] Tulving E. (1985). Memory and consciousness. *Can. Psychol.* 26 1–12 10.1037/h0080017

[B124] Ungerleider L. G. Mishkin M. (1982). “Two cortical visual systems,” in *Analysis of Visual Behavior*, eds Ingle D. J. Goodale M. A. Mansfield R. J. W. (Cambridge, MA: The MIT Press), 549–586

[B125] Unsworth N. Engle R. W. (2007). The nature of individual differences in working memory capacity: active maintenance in primary memory and controlled search from secondary memory. *Psychol. Rev.* 114 104–132 10.1037/0033-295X.114.1.104 17227183

[B126] Van Dijk H. Schoffelen J. M. Oostenveld R. Jensen O. (2008). Prestimulus oscillatory activity in the alpha band predicts visual discrimination ability. *J. Neurosci.* 28 1816–1823 10.1523/JNEUROSCI.1853-07.2008 18287498 PMC6671447

[B127] VanRullen R. Carlson T. Cavanagh P. (2007). The blinking spotlight of attention. *Proc. Natl. Acad. Sci. U.S.A.* 104 19204–19209 10.1073/pnas.0707316104 18042716 PMC2148268

[B128] VanRullen R. Koch C. (2003). Is perception discrete or continuous? *Trends Cogn. Sci.* 7 207–213 10.1016/S1364-6613(03)00095-0 12757822

[B129] VanRullen R. Reddy L. Koch C. (2005). Attention-driven discrete sampling of motion perception. *Proc. Natl. Acad. Sci. U.S.A.* 102 5291–5296 10.1073/pnas.0409172102 15793010 PMC555984

[B130] VanRullen R. Reddy L. Koch C. (2006). The continuous wagon wheel illusion is associated with changes in electroencephalogram power at ∼13 Hz. *J. Neurosci.* 26 502–507 10.1523/JNEUROSCI.4654-05.2006 16407547 PMC6674399

[B131] Varela F. J. Toro A. John E. R. Schwartz E. L. (1981). Perceptual framing and cortical alpha rhythm. *Neuropsychologia* 19 675–686 10.1016/0028-3932(81)90005-1 7312152

[B132] Venables P. H. (1960). Periodicity in reaction time. *Br. J. Psychol.* 51 37–43 10.1111/j.2044-8295.1960.tb00722.x 13841649

[B133] Vicario G. B. (2005). *Il Tempo. Saggio di Psicologia Sperimentale*. Bologna: Il Mulino

[B134] Wackermann J. Ehm W. (2006). The dual klepsydra model of internal time representation and time reproduction. *J. Theor. Biol.* 239 482–493 10.1016/j.jtbi.2005.08.024 16202427

[B135] Wearden J. H. (2001). “Internal clocks and the representation of time,” in *Time and Memory. Issues in Philosophy and Psychology* eds Hoerl C. McCormack T. (Oxford: Oxford University Press).

[B136] Wearden J. H. Edwards H. Fakhri M. Percival A. (1998). Why ‘sounds are judged longer than lights’: application of a model of the internal clock in humans. *Q. J. Exp. Psychol.* 51B, 97–120 10.1080/713932672 9621837

[B137] Wittmann M. (2011). Moments in time. *Front. Psychol.* 5:66 10.3389/fnint.2011.00066 PMC3196211 22022310

[B138] Wittmann M. (2013). The inner sense of time: how the brain creates a representation of duration. *Nat. Rev. Neurosci.* 14 217–223 10.1038/nrn3452 23403747

[B139] Wittmann M. Simmons A. N. Aron J. L. Paulus M. P. (2010). Accumulation of neural activity in the posterior insula encodes the passage of time. *Neuropsychologia* 48 3110–3120 10.1016/j.neuropsychologia.2010.06.023 20600186 PMC2933788

[B140] Wittmann M. Simmons A. N. Flagan T. Lane S. D. Wackermann J. Paulus M. P. (2011). Neural substrates of time perception and impulsivity. *Brain Res.* 1406 43–58 10.1016/j.brainres.2011.06.048 21763642 PMC3145046

[B141] Zakay D. Block R. A. (2004). Prospective and retrospective duration judgments: an executive-control perspective. *Acta Neurobiol. Exp.* 64 319–328 10.55782/ane-2004-1516 15283475

